# Plyometric jump training micro- and high-dose effects on amateur basketball players athletic performance: a randomized controlled trial

**DOI:** 10.3389/fphys.2025.1684022

**Published:** 2025-09-30

**Authors:** Bitai Wu, Baiming Zhang, Mingyue Yin, Kai Xu, Rodrigo Ramirez-Campillo, Sen Huang, Limingfei Zhou, Leijiao Yue, Jianxiong Li, Zhifeng Liu, Zhiquan Song, Boyi Zhang, Hengxian Liu, Long Li, Ken Zhang

**Affiliations:** ^1^ School of Physical Education, Changsha University of Science and Technology, Changsha, China; ^2^ School of Athletic Performance, Shanghai University of Sport, Shanghai, China; ^3^ Universidad de Tarapacá, Instituto de Alta Investigación, Sport Sciences and Human Performance Laboratories, Arica, Chile; ^4^ Universidad Andres Bello, Faculty of Rehabilitation Sciences, Exercise and Rehabilitation Sciences Institute, Santiago, Chile; ^5^ Universidad de Los Lagos, Department of Physical Activity Sciences, Osorno, Chile; ^6^ Hunan Institute of Sports Science, Changsha, China; ^7^ School of Strength and Conditioning Training, Beijing Sport University, Beijing, China; ^8^ Physical education and training Institute, Capital University of Physical Education and Sports, Beijing, China; ^9^ School of Physical Education and Sports Training, Hunan sports vocational college, Changsha, China; ^10^ School of Physical Education and Sports Science, Hengyang Normal University, Hengyang, China; ^11^ School of Physical Education, University of Science and Technology Beijing, Beijing, China; ^12^ Department of Molecular and Cellular Sports Medicine, Institute of Cardiovascular Research and Sports Medicine, German Sport University, Cologne, Germany

**Keywords:** plyometric exercise, human physical conditioning, muscle strength, team sports, resistance training, athletic performance, microdosing training

## Abstract

**Purpose:**

To compare the effects of a 4-week plyometric training program involving microdosing (MPT; four sessions per week, 400 total jumps) and highdosing (HPT; 2 sessions per week, 800 total jumps) on athletic performance in male amateur basketball players.

**Methods:**

Participants were randomly assigned to the MPT (n = 24), HPT (n = 23), or control (CTR; n = 22). The intervention training protocol involved 4 weeks, while the control group received no additional training. Twelve performance metrics were assessed pre- and post-intervention, including countermovement jump (CMJ), countermovement jump with arms (CMJA), 40 cm-drop jump (DJ), reactive strength index (RSI) and modified reactive strength index (RSImod), maximal isometric squat strength, isometric mid-thigh pull (IMTP) rate of force development (RFD), 10 m and 20 m sprint, T-test agility, 5-0-5 test agility, and endurance capacity (yo-yo intermittent recovery test-level 2 - YYIR2). Paired and independent sample t-tests were used to assess within- and between-group (with Bonferroni correction) differences. Effect sizes were calculated using Hedges’ g (*g*) to quantify the magnitude of training effects.

**Results:**

Compared to the control group, MPT improved CMJ (g = 0.66), RSI (g = 0.96), RSImod (g = 0.71), IMTP (g = 0.64), and 20 m sprint (g = 0.58), and HPT improved RSI (g = 1.08), RSImod (g = 0.88), IMTP (g = 0.64), 20 m sprint (g = 0.79). No differences were observed between MPT and HPT.

**Conclusion:**

Both MPT and HPT similarly enhance basketball players’ athletic performance. However, microdosing required reduced training volume to attain a similar magnitude of improvement, representing a time-efficient training alternative.

## 1 Introduction

Athletes comonlly face unforeseen constraints that may disrupt their training plans, more often related to time availability, which can hinder their pursuit of optimal performance. While traditional training models offer structured approaches to performance enhancement, they often require lengthy sessions and lack flexibility, making them less suitable for athletes with limited time or varying schedules (Bonder And Shim, 2022). To address these limitations, microdosing training emerged, aiming to retain the overall benefits of traditional training cycles (linear and nonlinear), involving an increase in the number of training sessions (e.g., per week), although reducing training session time to a minimum (e.g., minimal effective training time) (Comer et al., 2022). Shorter, more varied training sessions are often better received by athletes and may help mitigate the effects of training disruptions. This approach refines the training load into more frequent, smaller sessions within a given cycle (Afonso et al., 2022). Additionally, microdosing can serve as a compensatory training method for substitutes or players with reduced playing time (Vretaros, 2022). Regarding the practice of microtraining, which is similar to microdosing training, in physical fitness and athletic performance, it originated from military contexts (Bonder and Shim, 2022). Under the condition of equivalent total training volume, compared to longer, less frequent training sessions conducted over a week, shorter, high-frequency microtraining sessions did not yield any significant differences in strength and endurance outcomes (Kilen et al., 2015). However, Afonso et al. (2022) emphasized that microdosing should not simply be viewed as a repackaging of traditional distributed training. Instead, it should prioritize delivering the minimum effective dose through high-frequency training, with a total workload lower than that of conventional methods, while still promoting adaptation.

Current research suggests that the optimal microdosing frequency ranges from 2 to 4 sessions per week, each lasting 15–25 min (Comer et al., 2022; Vretaros, 2022), and that this approach is particularly effective for well-trained individuals (Bompa and Buzzichelli, 2019; Hoffman et al., 1990). Studies also recommend the use of compound, multi-joint movements to maximize efficiency within the limited training time. Given these time constraints, warm-ups and cooldowns are often omitted; instead, the initial set of formal exercises may function as a warm-up, while resistance training itself can incorporate stretching components (Bonder and Shim, 2022; Vretaros, 2022). Beyond improving athletic performance, microdosing also offers benefits in managing fatigue. By increasing the frequency of low-volume sessions, athletes are provided with more frequent opportunities for active recovery, thereby helping to reduce accumulated fatigue (Comer et al., 2022; Cuadrado-Peñafiel et al., 2023).

Plyometric training is widely utilized by athletes due to its unique muscle contraction patterns, particularly its emphasis on the stretch-shortening cycle. Its effectiveness in enhancing sports performance has been well-documented. In basketball, plyometric training has been shown to significantly improve vertical jump height, linear sprint speed, muscular strength, agility, and other performance-related attributes (Arazi and Asadi, 2011; Ramirez-Campillo et al., 2022; Ramírez-Delacruz et al., 2022; Zhou et al., 2024; Sáez-sáez de Villarreal et al., 2010). Additionally, it plays a crucial role in enhancing sport-specific skills in ball games (Ramírez-Campillo et al., 2015a).To maximize training outcomes, increasing the difficulty and volume of plyometric exercises—as well as incorporating progressive overload strategies such as resistance training—may help optimize athletic performance through physiological adaptation (Ramírez-Campillo et al., 2015a; Fatouros et al., 2025; Palma-Muñoz et al., 2021; Oliver et al., 2024). Moreover, combining vertical and horizontal plyometric exercises is more effective than using either type alone, leading to greater improvements in explosive power, balance, and intermittent aerobic capacity (Ramírez-campillo et al., 2015b).

However, a higher volume of resistance training does not necessarily lead to better outcomes. In a recent review on training volume in team sport athletes, Clemente et al. (Clemente et al., 2025) reported that high-volume training did not produce significantly greater benefits compared to low-volume training. Numerous experimental studies have similarly demonstrated no significant differences between low- and high-volume plyometric training in terms of their effects on body composition, vertical jump performance, strength, sprint speed, agility, endurance, and other physical attributes (Ramírez-campillo et al., 2015a; Palma-muñoz et al., 2021; Bianchi et al., 2019; Chaabene and negra, 2017; Liu et al., 2024). Furthermore, both low- and high-volume plyometric training have been shown to result in similar delayed declines in vertical jump ability among athletes (De Villarreal et al., 2008). These findings may highlight a key principle underlying microdosing plyometric training.

Currently, microdosing plyometric training remains in its early stages of development, primarily due to individual differences among athletes, variations across sports, and the cumulative load from conventional training programs (Liu et al., 2024). To date, only one study has examined the effects of microdosing plyometric training, at 50% of the original volume, on lower limb performance in football players. Existing research has primarily focused on its impact on vertical jump performance, speed, and power. However, its effects on endurance and change-of-direction ability represent important directions for future research on volume-based plyometric training (Afonso et al., 2022; Clemente et al., 2025). Additionally, the long-term effectiveness and sustainability of microdosing training (including post-training detraining effects), as well as its compatibility with progressive overload principles, warrant further investigation.

This study aims to examine the effects of microdosing and highdosing plyometric training on vertical jump performance, muscular strength, sprint speed, change-of-direction ability, and aerobic capacity in male amateur basketball players. Based on previous results (Liu et al., 2024), it was hypothesized that both microdosing and highdosing plyometric training would induce similar improvement magnitude in vertical jump performance, strength, sprint speed, change-of-direction ability, and aerobic capacity. Therefore, microdosing plyometric training will induce greater training efficiency (e.g., magnitude of improvement per jump).

## 2 Methods

### 2.1 Participants

A total of 77 male sophomore students majoring in basketball met the inclusion criteria and voluntarily participated in this study. The inclusion criteria were as follows: i) no participation in competitions or special training activities within the 4 weeks *prior* to the experiment, aside from regular coursework and the experimental intervention; ii) no history of sports-related injuries or illnesses within the past 6 months; iii) completion ≥70% of the planned training load (e.g., number of repetitions), with a rating of perceived exertion (RPE) score between 6 and 7 following each session (The RPE was measured after each session) (Gonçalo et al., 2025; Qu and Li, 2004). All participants were classified as Level 2 athletes based on their athletic qualifications (Mckay et al., 2022). To ensure allocation concealment, the 77 participants were randomly assigned to three groups and were informed of their respective training protocols during the intervention period.

**FIGURE 1:**
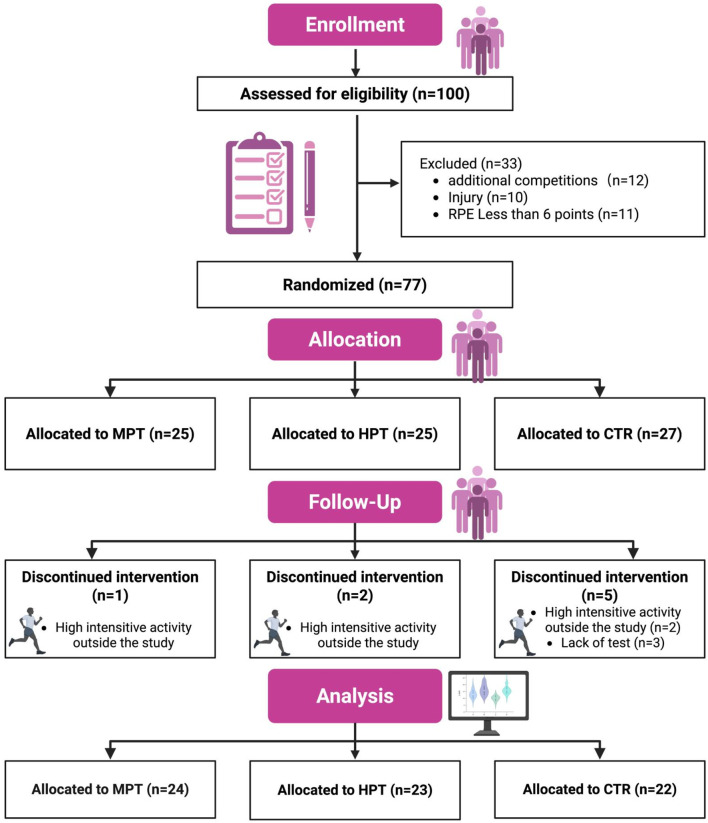
Participants inclusion design.

The experimental intervention was added to the participants’ regular academic coursework. The basketball and physical education courses were conducted by the same instructor, consisting of 400 min of basketball-specific practice and 270 min of coaching practice classes per week. Participants were instructed not to engage in any additional physical training beyond the scheduled coursework and experimental interventions. They were also required to complete a weekly physical activity log using the Additional Physical Activity Scale (Qu and Li, 2004). During the experiment, eight participants were excluded. Five were removed for reporting moderate to high-intensity physical activity outside the prescribed program, as recorded in their activity logs. Three others were excluded due to missing more than three training sessions for personal reasons, such as university-assigned travel or injuries sustained during the intervention. Ultimately, 69 participants completed the entire study. Participants were informed about the experimental procedures, including potential risks and benefits, and provided written informed consent prior to the study. The study was conducted in accordance with the Declaration of Helsinki and was approved by the Ethics Committee of ***BLIND FOR PEER-REVIEW*** University (2024433H) (Figure 1; Table 1).

**TABLE 1:** Participants baseline characteristics.

Parameter	MPT (n = 24)	HPT (n = 23)	CTR (n = 22)
Age (years)	20.41 ± 0.63	20.71 ± 1.07	20.52 ± 0.66
Height (cm)	180.81 ± 5.91	180.42 ± 6.39	181.18 ± 4.57
Body mass (kg)	80.35 ± 13.77	74.45 ± 10.65	72.67 ± 9.63
Body mass index (kg·m^-2^)	24.71 ± 3.39	22.83 ± 2.21	22.23 ± 2.68
Experience in basketball training (years)	3.78 ± 1.35	5.23 ± 1.70	4.52 ± 2.62
Experience in resistance training (years)	2.17 ± 1.17	4.00 ± 1.21	3.13 ± 1.36

Abbreviations: MPT, microdosing plyometric training; HPT, highdosing plyometric training; CTR, control group.

### 2.2 Experimental design

**FIGURE 2:**
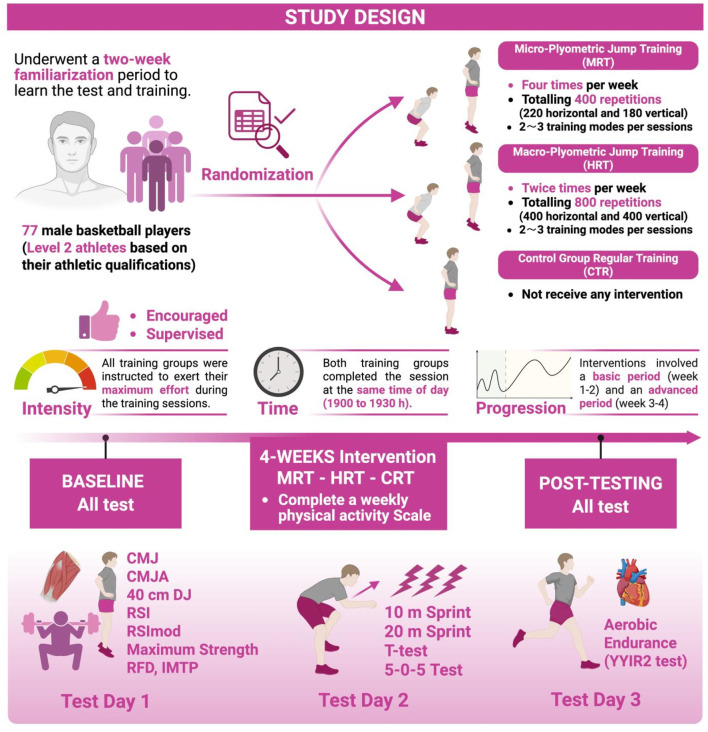
Schematic diagram of the study design.

None of the participants had prior experience with systematic plyometric training. Before the experiment, all participants underwent a 2-week familiarization period to learn the test and training procedures, ensuring they had sufficiently mastered the experimental protocol. Subsequently, using a randomization method generated in Microsoft Excel, participants were randomly assigned to the microdosing plyometric training [MPT], highdosing plyometric training [HPT], or control group [CTR] (Figure 2).

### 2.3 Training program

While the control group did not receive any supplementary training, both training groups completed the same jump exercises, with the same intensity (e.g., participants instructed to exert maximum effort-focus), at the same time of day (19:00 to 19:30), with differences only in total number of jumps after the 4 weeks of intervention (HPT = 400 jumps; MPT = 200 jumps), and content combination due to different weekly training frequency (Table 2).

**TABLE 2:** Intervention training protocols.

Week	Group	Monday	Tuesday	Wednesday	Thursday
1-2	MPT (172 jumps, 76 horizontal, 96 vertical)	Double-leg squat jump without arms (reverse) × 10, single-leg horizontal long jump × 12	Double-leg abdominal jump + long jump × 6, lunge vertical jump and swing leg alternation × 12	Single-leg vertical jump × 12, double-leg horizontal long jump twice × 5	Single-leg lateral jump × 10, 40 cm box DJ × 8
	HPT (344 jumps, 152 horizontal, 192 vertical)	No training	No training	Same as MPT on (Monday + Tuesday) × 2^a^	Same as MPT on (Wednesday + Thursday) × 2^a^
3-4	MPT (228 jumps, 124 horizontal, 104 vertical)	30 cm + 40 cm box DJ with 20 kg × 10, single-leg horizontal triple jump × 4	Single-leg lateral jump + single-leg 40 cm box vertical jump × 12, 15 cm box lunge vertical jump alternating with 20 kg × 10	Double-leg continuous hurdle vertical jump × 6, single-leg lateral jump + double-leg long jump × 10	Single-leg Bulgarian squat jump with 20 kg × 14, standing triple jump × 6
	HPT (456 jumps, 248 horizontal, 208 vertical)	No training	No training	Same as MPT on (Monday + Tuesday) × 2^a^	Same as MPT on (Wednesday + Thursday) × 2^a^

Abbreviations: DJ: drop jump, MPT: microdosing plyometric training, HPT: highdosing plyometric training.

^a^ Exercises content combination varied slightly. For example, compared to MPT, during session 1 of the first 2 weeks, the HPT, group completed double-leg squat jumps with arms (reverse) × 10, followed by lunge vertical jump and swing leg alternation × 12.

Interventions involved basic (week 1–2) and advanced training periods (week 3–4) (Ramírez-Campillo et al., 2015a). Weeks 1-2 involved horizontal, vertical, unilateral, and bilateral bodyweight-based jumps (e.g., double-leg squat jump without arms, single-leg horizontal long jump, single-leg lateral jump), with focus on execution technique. In week 3-4 some loaded (external load) jumps were included, the number of total repetitions increased, as well as the difficulty (*intensity*) of some jumps. The recovery time between exercises sets was 2–3 min. Before training sessions ∼5 min of warm-up was included, involving dynamic stretching (e.g., the greatest stretch, hamstring stretch), calf raises, single-leg pull-back superman, high leg lifts, back kicks, small-step sprints (Bonder and Shim, 2022).

### 2.4 Test methods and procedures

Basketball is a sport with various physical performance characteristics, and its performance is not determined by a single physical quality alone. It is the result of a combination of multiple components (Taylor et al., 2017). Therefore, we set up 12 relevant test indicators, which include (i) jump-related measures, (ii) strength-related measures, (iii) sprint performance, (iv) change-of-direction/agility, and (v) aerobic capacity. This comprehensive exploration is the strength of this study, which helps us understand whether plyometric jump training (PTJ) is effective and also enables us to systematically reveal the specific differences in performance caused by MPT and HPT. Before and after the training intervention, participants’ athletic performance was assessed. On the first day, the CMJ, CMJA, 40 cm DJ, RSI, RSImod, maximum strength, and the rate of force development (RFD) during the IMTP test were completed. On the second day, participants underwent the 10 m sprint, 20 m sprint, T-test, and 5-0-5 test. The third day focused on the YYIR2 test (interval running endurance). Before all testing sessions, participants were required to complete a standardized warm-up consisting of 5 min of jogging followed by 5 min of dynamic stretching. Additionally, participants completed specific warm-up attempts before each test. Participants were instructed to refrain from consuming alcohol 48 h before the test and to avoid caffeine or hormone-related nutritional supplements for 8 h before testing. Due to limited resources, in this experiment, the test subjects did not use a blind method.

#### 2.4.1 Vertical CMJ

The CMJ height was measured using a portable dual force platform and its associated software (Kunwei, Model: KWYp-Fp6035-7K, Shanghai, China), H = v^2^/2 g (H; height, v; Take-Off velocity, g; 9.8 m/s^2^) (Mao et al., 2024). Participants performed the CMJ test, keeping their hands on their hips throughout the jump. They were asked to jump as high as possible while keeping their lower limbs fully extended during the take-off phase. Each participant completed three attempts, with 1 min of rest between trials, and the best score was recorded.

#### 2.4.2 Vertical CMJA

Distinguishing from CMJ and CMJA, they are not merely tests for lower limb explosive power, but are also used to assess overall coordination and overall explosive power of the entire body. They are one of the tests that are more closely related to the specific sport (Lees et al., 2004). Participants performed the vertical CMJA test, with the same protocol used for the vertical CMJ test, although participants were allowed to swing their arms to assist the vertical jump during the CMJA, and the depth of each jump was self-selected.

#### 2.4.3 Vertical 40 cm DJ

From a 40 cm box (Ramirez-Campillo et al., 2018), participants completed three standard drop jumps followed by vertical jumps with arm swing. Participants were instructed to jump as quickly and as high as possible. The jump height, RSI, and RSImod were measured and/or calculated using a force platform (Kunwei, Model: KWYp-Fp6035-7K, Shanghai, China). It is worth noting that although accomplished through the same project, they respectively reveal different jumping abilities. The research indicates that RSI is typically used to assess the ability of a fall jump, while RSImod is more commonly used to measure the ability of a backward jump from the ground (Louder et al., 2021).

#### 2.4.4 Maximum strength

Participants performed an isometric back squat maximum strength test on a force platform (Kunwei, Model: KWYp-Fp6035-7K, Shanghai, China). After the standardized warm-up, participants completed a specific warm-up including 2–3 sets of back squats at 40%–60% of their perceived 1RM. Then, the isometric back squat was performed for 5 s on the force platform. Participants completed three trials, with 2 min of rest between trials. The highest strength value was recorded as the test score.

#### 2.4.5 The IMTP test for RFD

Prior to testing the barbell was positioned midway between the knee and hip joints, adjusted to the participant’s comfort level. Participants used an overhand grip and were instructed to pull the barbell upward as hard and fast as possible, maintaining maximum effort for 5 s. Each participant completed three attempts, with 2 min of rest between trials. Peak RFD at 50, 100, 150, 200, 250, and 300 m were recorded by a force plate (Kunwei, Model: KWYp-Fp6035-7K, Shanghai, China) (Aagaard et al., 2002).

#### 2.4.6 Linear sprint performance

In a standard wooden-floor gymnasium, participants performed three 10 m sprints and then three 20 m sprints, at maximal running velocity, with 1 min or 2 min of rest between 10 m and 20 m trials, respectively. Times were measured with two pairs of photoelectric tubes (SmartSpeed, Fusion Sport, Queensland, Australia; accuracy of 0.001 s).

#### 2.4.7 Non-reactive agility T-test

As previously outlined (Munro et al., 2011), participants were instructed to complete the agility course as fast as possible, requiring acceleration, deceleration, COD, involving forward, backward, and lateral displacement, always facing forward. Participants completed three valid maximal trials, with 2 min of rest between trials. The time needed to complete the test was used as a performance outcome, and was measured with two pairs of photoelectric tubes (SmartSpeed, Fusion Sport, Queensland, Australia; accuracy of 0.001).

#### 2.4.8 Non-reactive agility 5-0-5 test

As previously described, participants completed three maximal trials, as fast as possible, with 2 min of rest between trials. The time needed to complete the test was used as a performance outcome and was measured with two pairs of photoelectric tubes (SmartSpeed, Fusion Sport, Queensland, Australia).

#### 2.4.9 Endurance capacity

Aerobic Endurance (YYIR2): Research has demonstrated that the YYIR2 test is an effective method for assessing both aerobic and anaerobic interval capacity (KRUSTRUP et al., 2006). During the test, participants perform a 20-m round-trip run based on audio prompts. After completing each lap, there is a 10-s rest interval, during which the athlete can jog or relax in the 2 × 5-m area behind the starting point. The test ends if the athlete fails to meet the required pace twice. The total distance completed in the test is then used in the following formula to calculate maximum oxygen uptake: YYIR2 VO^2^max (mL*kg^-1^*min^-1^) = IR2distance (m) × 0.0136 + 45.3.

### 2.5 Statistical analysis

Blinded statistical analyses were performed by a third researcher, unaware of experimental group assignments. All data are presented as mean ± standard deviation (SD). An *a priori* power analysis was conducted using G*Power (version 3.1.9.7) (Faul et al., 2007) to determine the minimum sample size required to adequately test our primary hypotheses. The analysis was set for an independent-samples *t*-test, with a conventionally accepted effect size of *d* = 0.80 (representing a “large” effect based on Cohen’s criteria and relevant literature in athletic performance) (Muller, 1989), an alpha level (α) of 0.05, and a desired statistical power (1 -β) of 0.80. The results indicated that a total sample size of N = 42 (or n = 21 per group) would be sufficient to detect the hypothesized effects. Therefore, our study was adequately powered to detect significant between-group differences on the primary outcome measures. Data normality was verified using the Shapiro–Wilk test. Within-group pre-post intervention changes were assessed with a paired sample *t*-test. Between-groups differences were assessed with an independent sample *t*-test, and to control for multiple comparisons and reduce the risk of type I error, the Bonferroni correction was applied. Statistical significant results were set at *p* < 0.05, and at *p* < 0.01 for highly significant (Discovering statistics using IBM SPSS, 2017-10). Effect sizes (ES) were calculated using Hedges’ *g*, interpreted as follows: trivial (<0.2), small (0.2–0.6), moderate (>0.6–1.2), large (>1.2–2.0), and very large (>2.0) (Hopkins et al., 2009). All statistical analyses were performed using R software (version 4.3.0).

## 3 Results

The reliability of the outcome measures is provided in Table 3.

**TABLE 3:** Outcomes measure’s reliability.

Category	Outcome	Intraclass correlation coefficient (95% CI)	Coefficient of variation (%)
Vertical jump	CMJ	0.97 (0.96–0.98)	2.42%
	CMJA	0.98 (0.97–0.98)	2.06%
	40 cm DJ	0.92 (0.89–0.94)	4.37%
	RSI	0.90 (0.87–0.92)	6.80%
	RSImod	0.91 (0.88–0.93)	7.83%
Strength	Maximum strength	0.92 (0.90–0.94)	5.17%
	IMTP-50 m	0.45 (0.32–0.57)	30.47%
	IMTP-100 m	0.47 (0.34–0.60)	24.02%
	IMTP-150 m	0.57 (0.45–0.68)	20.00%
	IMTP-200 m	0.69 (0.59–0.78)	14.74%
	IMTP-250 m	0.68 (0.58–0.77)	12.62%
	IMTP-300 m	0.65 (0.54–0.74)	12.28%
Sprint/Change-of-direction	10 m	0.78 (0.73–0.83)	2.65%
	20 m	0.90 (0.87–0.93)	1.47%
	T-test	0.90 (0.87–0.92)	2.09%
	5-0-5	0.76 (0.70–0.82)	2.91%
Endurance capacity	YYIR2	NA	NA

Abbreviations: CI, confidence interval; CMJ, countermovement jump; CMJA, countermovement jump with arms; DJ, drop jump; IMTP, isometric mid-thigh pull; RSI, reactive strength index; RSImod, modified reactive strength index; YYIR2, yoyo intermittent recovery test-level 2).

### 3.1 Vertical jump performance

**FIGURE 3:**
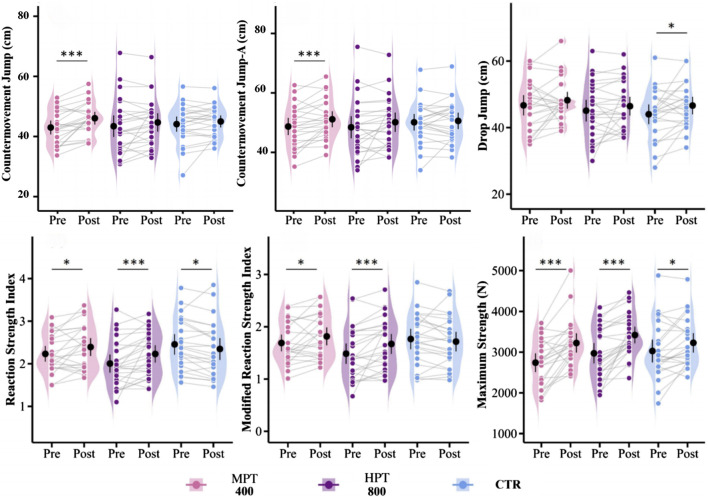
Effects of MPT and HPT on jump performance. Note: Violin plots with individual data points (Pre vs. Post) showing changes in vertical jump performance across three groups: microdosing plyometric training (MPT), highdosing plyometric training (HPT), and control group (CTR). CMJ; CMJ without swing arm, CMJA; CMJ with the swing arm. Significant within-group pre–post differences and between-group comparisons are marked. *p < 0.05, **p < 0.01, ***p < 0.001.

Compared to baseline, the microdosing training group showed a significant improvement in CMJ (g = 0.87, p < 0.01) and CMJA (g = 0.69, p < 0.01), as well as a small but significant improvement in RSI (g = 0.47, p < 0.05) and RSImod (g = 0.45, p < 0.05) in Figure 3. The highdosing training group exhibited a very significant improvement in RSI (g = 0.59, p < 0.01) and RSImod (g = 0.60, p < 0.01). The control group also showed a small significant effect size in 40 cm DJ (g = 0.50, p < 0.05) and RSI (g = 0.49, p < 0.05). Post hoc comparisons revealed that the microdosing group had a moderately significant improvement in CMJ (g = 0.66, p < 0.05), a very significant improvement in RSI (g = 0.96, p < 0.01), and a moderately significant benefit in RSImod (g = 0.71, p < 0.05) compared to the control group. The highdosing had a very significant improvement in RSI (g = 1.08, p < 0.001) and RSImod (g = 0.88, p < 0.01) compared to the control group. Additionally, the inter-group comparisons showed no significant difference between the microdosing and highdosing in the improvement of RSI (g = 0.18, p = 0.52) and RSImod (g = 0.21, p = 0.47).

### 3.2 Strength

**FIGURE 4:**
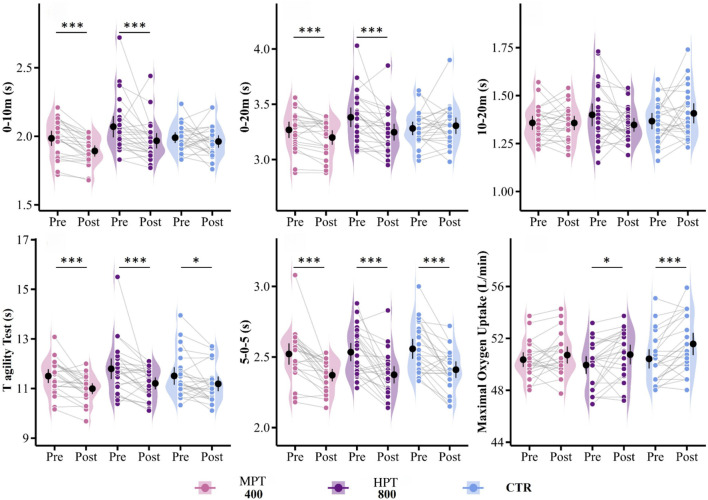
Effects of MPT and HPT on strength. Note: Violin plots with individual data points (Pre vs. Post) showing changes in strength across three groups: microdosing plyometric training (MPT), highdosing plyometric training (HPT), and control group (CTR). Significant within-group pre–post differences and between-group comparisons are marked. *p < 0.05, **p < 0.01, ***p < 0.001.

Compared to baseline, the microdosing training group showed a highly significant improvement in maximum strength (g = 0.96, p < 0.001) and a moderately significant improvement in each phase of the IMTP test (g = 0.60–0.98, p < 0.01) in Figure 4. The highdosing training group also demonstrated a highly significant improvement in maximum strength (g = 1.13, p < 0.001), as well as a highly significant improvement in each phase of the IMTP (g = 0.78–1.16, p < 0.001). In the control group, a small significant effect was observed in the improvement of maximum strength (g = 0.50, p < 0.05), and a significant improvement was noted in each phase of the IMTP (g = 0.52–0.78, p < 0.05). Post hoc comparisons revealed that the microdosing training group had a moderately significant effect on the improvement of maximal strength compared to the control group (g = 0.64, p < 0.05), while the highdosing training group also showed a significant effect on the improvement of maximal strength (g = 0.64, p < 0.05). There was no significant difference between the two training groups in the improvement of maximal strength (g = 0.07, p = 0.79).

### 3.3 Sprint and change of direction performance

**FIGURE 5:**
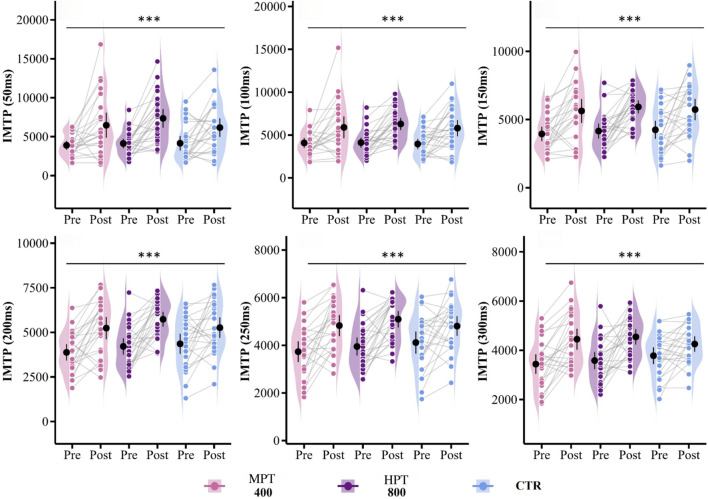
Effects of MPT and HPT on linear and non-linear sprint, and endurance performance. Note: Violin plots with individual data points (Pre vs. Post) showing changes in strength across three groups: microdosing plyometric training (MPT), highdosing plyometric training (HPT), control group (CTR), and isometric mid-thigh pull (IMTP). Significant within-group pre–post differences and between-group comparisons are marked. *p < 0.05, **p < 0.01, ***p < 0.001.

In the comparison between groups before and after the intervention, we found that, compared to baseline, the microdosing training group showed very significant improvements in the 10 m sprint (g = 1.2, p < 0.001), 20 m sprint (g = 0.61, p < 0.01), T-test (g = 0.88, p < 0.001), and 5-0-5 test (g = 0.97, p < 0.001) in Figure 5. The highdosing training group also exhibited significant improvements in the 10 m sprint (g = 0.54, p < 0.05), 20 m sprint (g = 0.65, p < 0.01), T-test (g = 0.58, p < 0.01), and 5-0-5 test (g = 0.90, p < 0.001), with varying degrees of significance. Additionally, the control group demonstrated significant improvements in the T-test (g = 0.48, p < 0.05) and 5-0-5 test (g = 0.93, p < 0.001). Post hoc comparisons revealed that, compared to the control group, the microdosing training group showed a small but significant effect on the improvement of the 20 m sprint (g = 0.58, p < 0.05). The highdosing group exhibited significant improvements in the 20 m sprint (g = 0.79, p < 0.01). Furthermore, no significant difference was found between the microdosing and highdosing training groups in the improvement of the 20 m sprint (g = 0.39, p = 0.17).

### 3.4 Endurance capacity

Compared to baseline, the highdosing training group (g = 0.48, p < 0.05) and the control group (g = 0.78, p < 0.01) exhibited significant improvements in YYIR2. However, *post hoc* comparisons revealed no significant differences between the groups.

## 4 Discussion

This study examined the effects of 4 weeks of microdosing and highdosing plyometric training on athletic performance in male amateur basketball players. Both training methods improved jump performance, lower-limb strength, and sprint ability, with comparable outcomes despite the lower volume in the microdosing group. This suggests that even brief, low-dose protocols can be effective in enhancing key athletic qualities. However, neither approach appeared to impact change of direction, explosive force output, or aerobic capacity, indicating that these attributes may require different or longer-term interventions.

### 4.1 Vertical jump ability

Considering that vertical jump ability may play a crucial role in basketball (Ziv and Lidor, 2010), the increased CMJ in the MPT group is a relevant novel finding. Jump training may be related to multiple physiological-biomechanical variables associated with jumping, including (but not limited to) increased motor unit firing rate and/or recruitment (e.g., increased skeletal muscle EMG activity), increased RFD, increased maximal strength (Markovic and Mikulic, 2010). Of note, while the MPT group improved CMJ, the HPT did not. If physiological and/or biomechanical variables associated jump performance showed a different adaptation pattern in MPT compared to HPT is currently unclear since our study did not included phyiological-biomechanical measurements. Therefore, it seems that the lower number of jumps per session (400 jumps) and higher training frequency (4 sessions per week) of the MPT compared to the HPT yielded greater training efficiency (i.e., unit of improvement in CMJ per training jump), in line with previous finding (Ramirez-Campillo et al., 2022; Ramírez-Delacruz et al., 2022). Indeed, a systematic review reported greater jump performance improvements in basketball players with greater jump training frecuency (Ramirez-Campillo et al., 2022). Nonetheless, future studies are advised to elucidate the underlying biological mechanisms related to the apparently greater training efficiency of MPT compared to HPT in basketball players.

Additionally, the difference between CMJ and CMJA was attributable to arm swing. Despite the subjects having an average of 4.5 years of experience, there were noticeable differences in arm swing technique among individuals, and even within the same subject, the application of arm swing fluctuated significantly between tests. This could explain why CMJA did not show significant improvements. The lack of positive effects in the HPT group remains unclear. We speculate that the low frequency of stimulation (only eight sessions in the HPT group compared to 16 in the MPT group) may have been insufficient to induce improvements in vertical jump performance, especially considering that most subjects had not undergone systematic plyometric training before. Additionally, the higher training volume (800 jumps in the HPT group, double that of the MPT group) combined with fatigue accumulation from regular basketball training could have contributed to the lack of improvement in vertical jump performance (De Villarreal et al., 2008; González-Badillo et al., 2006). The students also had daily theoretical and practical courses during the 4-week intervention, which likely contributed to their fatigue. In a fatigued state, large-volume plyometric training may fail to yield positive effects. Moreover, the HPT group’s single training sessions lasted over 30 min, three times longer than those of the MPT group. Although we provided consistent verbal encouragement and monitored training intensity, the extended training duration likely caused fatigue that affected the participants’ motivation and training effectiveness, which may explain the lack of significant improvement. In the 40CMDJ test, there were no significant changes before and after the intervention in either training group, while the control group showed significant improvements. We believe this may be attributed to the conventional basketball training regimen.

Previous systematic reviews have highlighted plyometric training as a key method for effectively improving RSI, with its effects being more pronounced in adult athletes compared to adolescents (Rebelo et al., 2022; Ramirez-Campillo et al., 2023). The findings of this study not only support this perspective but also reveal that plyometric training at different volumes and frequencies (i.e., MPT group and HPT group) produced similar effects on both RSI and RSImod (the modified Reactive Strength Index). This outcome aligns with the observations of Liu et al. (Ramírez-Campillo et al., 2015a; Liu et al., 2024). Following the recommendations of Campillo et al., we measured the RSI using a 40 cm medium-height jump box drop jump (Ramirez-Campillo et al., 2018). RSI serves as an indicator to evaluate an athlete’s ability to rapidly generate force through a swift transition from eccentric to concentric muscle contraction (Jarvis et al., 2022). From a certain perspective, RSI size is positively correlated with an athlete’s performance and level of proficiency (Jarvis et al., 2022; Lockie et al., 2011). Furthermore, RSImod, which has broader application and greater reliability, is also widely used (Suchomel et al., 2015). Notably, due to the specificity of these two outcome indicators, RSI (typically measured using drop jumps) and RSImod (typically measured using ground jumps such as CMJA and SJ) are related but not interchangeable (Louder et al., 2021).

Therefore, in this experiment, the results of plyometric training on the RSI were reported more comprehensively by testing two outcome indicators (Louder et al., 2021). Plyometric jump training primarily enhances the rate of force development through the stretch-shortening cycle (SSC). It supports concentric performance through the principle of eccentric lengthening. Following the principle of training specificity, plyometric training is highly effective for improving RSI through neuromuscular adaptation (Markovic and Mikulic, 2010). However, the increase in RSI may also be associated with improved tendon stiffness. Indeed, structural changes in muscles caused by plyometric training, including increased muscle thickness, muscle fascicle angle, muscle bundle length, and tendon stiffness, collectively may contribute to improved RSI and RSImod (Ramírez-Delacruz et al., 2022). Additionally, RSI has a strong correlation with speed, acceleration, agility, and maximal strength (Rebelo et al., 2022), in line with our results, as discussed in the following paragraph.

### 4.2 Strength

Plyometric training is widely recognized for its ability to enhance maximal strength. It not only increases maximal isometric strength but also contributes to improvements in muscular endurance and other physical qualities (Ramirez-Campillo et al., 2022; Ramírez-Delacruz et al., 2022; Sáez-Sáez De Villarreal et al., 2010; Fatouros et al., 2025; Singh et al., 2021). A notable finding of this study is that the improvement in maximal strength observed in the MPT group was comparable to that of the HPT group. The observed strength gains from plyometric training may be attributed to neural adaptations, including increased motor unit firing rate, synchronization, excitability, and enhanced efferent motor drive (Markovic and Mikulic, 2010). Structural adaptations, such as muscle hypertrophy, increased muscle thickness, and fascicle lengthening, may also contribute to these improvements (Ramírez-Delacruz et al., 2022). Previously, low-volume plyometric training induced similar improvements in maximal strength as high-volume protocols (De Villarreal et al., 2008), a finding further supported by the present study.

However, some meta-analyses have presented contrasting conclusions, suggesting that plyometric training does not significantly enhance maximal strength (Oliver et al., 2024). Upon careful examination, we found that the two meta-analyses reporting no significant effects of plyometric training on maximal strength were primarily based on studies involving minors. The reported outcomes may have been influenced by factors such as the type of maximal strength testing employed, the specific plyometric training protocols used, and the participants’ stage of growth and development. Therefore, these findings do not compromise the validity of our results. In the inter-group comparison, following the 4-week plyometric training intervention, we also did not observe a significant improvement in RFD across different time intervals. This may be attributed to the testing methods used. Research on the impact of plyometric training on RFD remains limited. While some studies have indicated that the isometric mid-thigh pull (IMTP) is a reliable and accurate laboratory-based method for assessing peak force (PF) and RFD across various time domains, and that RFD values obtained via IMTP are closely associated with key athletic abilities such as speed, agility, weightlifting, vertical jumping, and sport-specific performance (Townsend et al., 2019; Mangine et al., 2016), the present study did not find significant changes—potentially due to the short intervention period or training specificity.

However, IMTP is a technically demanding movement derived from Olympic weightlifting. Its requirements for individualized joint angles and force application patterns pose practical challenges during experimental testing. Although the participants in this study had an average of 3.1 years of resistance training experience and had completed two theoretical and practical courses prior to testing, many still produced missing or invalid data due to improper execution during the IMTP test. Therefore, we recommend ensuring that subjects are thoroughly familiar with the movement technique and capable of performing it correctly before data collection. In the within-group analysis, we also found that the control group demonstrated a significant improvement in RFD across all time intervals. This may be attributed to the extensive running and jumping activities regularly included in standard basketball training, which can contribute to enhanced explosive power (Carrera, 2024). This factor may also explain the absence of significant between-group differences in RFD outcomes.

### 4.3 Sprint and change of direction ability

Plyometric training has been shown to effectively improve short-distance linear sprint performance in basketball and football players (Ramirez-Campillo et al., 2022; Moran et al., 2021). In this experiment, we unexpectedly found that the MPT group also significantly improved their 20-m sprint performance, with no significant difference observed between the two experimental groups. This finding supports previous research suggesting that plyometric training, regardless of volume and frequency, can yield similar improvements in sprinting performance (Chaabene and Negra, 2017; Liu et al., 2024; De Villarreal et al., 2008). Hakkinen et al. suggested that such improvements in sprinting may result from enhanced neuromuscular activation, specifically an increase in the number of activated motor units, higher firing frequency, and altered motor unit recruitment patterns (primarily in fast-twitch muscle fibers) (Häkkinen et al., 1986). This may explain the observed improvement in linear sprint performance following plyometric training. Furthermore, the improvements in RSI and maximal strength mentioned earlier could contribute to reduced ground contact time and enhanced explosive power, which are essential for faster sprints. In addition to muscle adaptation through plyometric training, the combination of horizontal jump training and resistance-based plyometric exercises in this experiment may also have played a significant role. Studies have shown that training programs emphasizing horizontal acceleration—such as sprint-specific plyometric exercises and horizontal displacement jumps—can notably improve sprint times (Wilson et al., 1993). Additionally, Oliver et al. highlighted that combining plyometric training with resistance training provides optimal benefits for sprint performance (Oliver et al., 2024). The integration of both training methods in this study further supports this previous research.

Regarding the ability to change direction, plyometric training, through its focus on eccentric movement, deceleration, and braking ability, as well as the enhancement of neuromuscular recruitment, can effectively improve an athlete’s agility and change of direction (Chaabene et al., 2018). Although no significant difference in change of direction ability was observed between the plyometric training groups and the control group, we were surprised to find that the control group also demonstrated a significant improvement in change of direction performance (T-test: p < 0.05, g = 0.48; 5-0-5 test: p < 0.01, g = 0.93). This suggests that the 4-week conventional basketball course itself led to a notable increase in change of direction ability, which may explain this result. Moreover, in some previous reviews (Ramirez-Campillo et al., 2022; Zhou et al., 2024), we observed that the subjects were adolescents (<18 years old), in contrast to the college-aged basketball players in our study (Ramírez-Campillo et al., 2015a; Palma-Muñoz et al., 2021; Chaabene and Negra, 2017). Additionally, our review of the literature revealed that Asadi et al. reported that enhanced training with an average frequency of 12–24 sessions and a total volume of 800–1,200 repetitions was most effective in improving change of direction ability (Asadi et al., 2016). Therefore, it is likely that the frequency and volume of training in this study contributed to the lack of significant improvement in this area.

### 4.4 Endurance capacity

The effect of plyometric training on the aerobic and anaerobic capacity of ball players has been a key area of interest in previous studies (Ramirez-Campillo et al., 2022; Liu et al., 2024). Studies have shown that for basketball players, YYIR2 is one of the effective methods for measuring VO2max. It has a significant correlation with the directly measured VO2max, and it also saves considerable costs (Gürses et al., 2018). The results of this study show that only the highdosing group showed a significant difference in the two intervention groups. The underlying mechanism behind this phenomenon is unknown to us. Although we found in studies on football players that enhanced training significantly improved aerobic capacity (Ramírez-Campillo et al., 2015a). Furthermore, we observed a significant improvement in aerobic capacity in the control group before and after the experiment, which may have been influenced by regular basketball training, potentially confounding the comparison results between the experimental groups.

### 4.5 Potential limitations

A 4-week training intervention (with 8–16 sessions) should be considered as an acute application of plyometric training Therefore, we recommend that future research on microdosing plyometric training focus on long-term effects. In our analysis, we observed significant improvements in many test indicators in the control group, which may have influenced the comparison between the two intervention groups. We are not clear about the underlying mechanism of this result. Although we used the physical activity scale to take into account the additional physical activity, the limited resources prevented us from effectively monitoring the load of the basketball technical training sessions. The numerous explosive, agility, and endurance exercises included in the basketball training sessions are likely to be the potential reasons for the partial improvement observed in the control group (Santos and Janeira, 2011). We suggest that future studies monitor and record the impact of regular basketball technical training on experimental interventions. The observed improvements in CMJ following MPT may be indirectly related to mechanisms such as increased motor unit recruitment, improved neuromuscular coordination, and more effective utilization of stretch–shortening cycles, as suggested by previous studies (Liu et al., 2024). However, these pathways were not directly assessed in the present study and should be empirically verified in future mechanistic investigations. During the test, due to the limited conditions and training time, we only monitored the load responses generated during training verbally, including RPE and so on. We suggest that in future research, this data be recorded in real time. Additionally, the reliability of certain outcome measures (e.g., IMTP at 50 m: ICC = 0.45, CV = 30.5%) was relatively low, which may have masked true intervention-induced changes and should be interpreted with caution. Finally, as this study is the first to examine the effects of microdosing plyometric training on basketball players, and given that the optimal training frequency and minimal dose for microdosing have not been established, we propose that future research explore the effects of microdosing plyometric training with higher frequencies (>4 times/week) and lower volumes (<100 jumps/week) on the physical characteristics and sports performance of basketball students, as well as its impact on fatigue.

### 4.6 Practical application

Although the minimum effective dose of microdosing training for basketball is still unclear, several factors may contribute to this uncertainty, including the cumulative stimulation from basketball games or training, fatigue and recovery mechanisms, and individual differences within sports populations. However, due to the increasing demands of basketball schedules, work conflicts, and variations in fatigue levels among players, investigating the optimal effective stimulation from microdosing training, characterized by low volume and high frequency, holds significant research potential and practical application value. This study demonstrates that during basketball training, lower doses and higher frequencies of plyometric training can yield the same benefits as highdosing training. This finding raises important questions for coaches about the necessity of increasing athletes’ physical training loads, particularly since basketball players already face a substantial, multi-faceted training load in their technical sessions. This experiment offers new insights for determining the minimum effective dose needed to improve performance while reducing the risk of fatigue-related injuries caused by overtraining.

## 5 Conclusion

This study demonstrates that 4 weeks of microdosing plyometric training is an efficient, time-saving, and low-volume strategy to improve countermovement jump height, lower-limb strength, and linear sprint performance in male amateur basketball players. Notably, the physical performance gains observed in the microdosing group were comparable to those in the highdosing training group, despite the substantially lower training volume. However, under the present protocol, no significant improvements were found in change of direction performance, rate of force development, or aerobic capacity. Considering the real-world constraints in basketball training environments, we recommend that athletes, coaches, and practitioners incorporate microdosing plyometric training into regular training routines as a practical and evidence-based alternative to higher-volume interventions for enhancing key physical capacities.

## Data Availability

The original contributions presented in the study are included in the article/Supplementary Material, further inquiries can be directed to the corresponding authors.
