# Supplementary material for: Plyometric jump training micro- and high-dose effects on amateur basketball players athletic performance: a randomized controlled trial
Source: Front Physiol. 2025 Sep 30;16:1684022. doi: 10.3389/fphys.2025.1684022 (PMC12518302; doi:10.3389/fphys.2025.1684022)
Supplement: Supplementary file 1 [file Table1.docx]

**Supplement 1**

| **Table supplementary 1.** Pre- and post-training changes in vertical jump and lower-limb neuromuscular performance across intervention groups. | | | | | |
| --- | --- | --- | --- | --- | --- |
|  | **Baseline** | **After training** | **p-value*** | **p.adj.signif** | **Hedges´ *g*** |
| CMJ (cm) |  |  |  |  |  |
| CTR | 44.11 ± 6.33 | 45.00 ± 4.97 | 0.173 |  | 0.28 |
| MPT | 42.99 ± 5.59 | 46.07 ± 5.18 | **<0.001** | *** | 0.8727593 |
| HPT | 43.42 ± 9.15 | 44.67 ± 7.80 | 0.162 | ns | 0.2789222 |
| **CMJA (cm)** |  |  |  |  |  |
| CTR | 50.12 ± 6.97 | 50.59 ± 6.92 | 0.527 | ns | 0.1266469 |
| MPT | 48.70 ± 7.08 | 51.14 ± 6.58 | **0.002** | ** | 0.685696 |
| HPT | 48.44 ± 9.50 | 50.14 ± 8.30 | 0.114 | ns | 0.3172912 |
| **40CM DJ (cm)** |  |  |  |  |  |
| CTR | 44.00 ± 7.39 | 46.62 ± 6.62 | **0.019** | * | 0.4956525 |
| MPT | 46.70 ± 7.42 | 48.22 ± 6.19 | 0.216 | ns | 0.2560786 |
| HPT | 45.08 ± 8.28 | 46.43 ± 7.15 | 0.236 | ns | 0.2349319 |
| **RSI** |  |  |  |  |  |
| CTR | 2.46 ± 0.60 | 2.35 ± 0.64 | **0.020** | * | -0.4937761 |
| MPT | 2.23 ± 0.45 | 2.39 ± 0.51 | **0.029** | * | 0.4684085 |
| HPT | 2.00 ± 0.54 | 2.23 ± 0.52 | **0.006** | ** | 0.5879112 |
| **RSImod** |  |  |  |  |  |
| CTR | 1.77 ± 0.49 | 1.72 ± 0.47 | 0.275 | ns | -0.2204638 |
| MPT | 1.69 ± 0.39 | 1.82 ± 0.42 | **0.034** | * | 0.4536254 |
| HPT | 1.48 ± 0.50 | 1.67 ± 0.49 | **0.005** | ** | 0.5951464 |
| Maximum strength (N) |  |  |  |  |  |
| CTR | 3027.87 ± 698.00 | 3228.50 ± 596.08 | **0.018** | * | 0.5036508 |
| MPT | 2741.38 ± 563.99 | 3224.28 ± 582.86 | **<0.001** | **** | 0.9584767 |
| HPT | 2971.07 ± 636.95 | 3420.07 ± 520.66 | **<0.001** | **** | 1.1327464 |
| **0-10 m (s)** |  |  |  |  |  |
| CTR | 1.99 ± 0.10 | 1.96 ± 0.11 | 0.347 | ns | -0.1891517 |
| MPT | 1.99 ± 0.13 | 1.89 ± 0.10 | **<0.001** | **** | -1.2033842 |
| HPT | 2.07 ± 0.20 | 1.97 ± 0.14 | **0.010** | ** | -0.544288 |
| **0-20 m (s)** |  |  |  |  |  |
| CTR | 3.28 ± 0.15 | 3.31 ± 0.19 | 0.554 | ns | 0.1183754 |
| MPT | 3.27 ± 0.18 | 3.20 ± 0.16 | **0.006** | ** | -0.614307 |
| HPT | 3.38 ± 0.22 | 3.25 ± 0.19 | **0.003** | ** | -0.6458693 |
| **T-test (s)** |  |  |  |  |  |
| CTR | 11.51 ± 0.88 | 11.19 ± 0.74 | **0.024** | * | -0.4753753 |
| MPT | 11.50 ± 0.68 | 10.99 ± 0.54 | **<0.001** | *** | -0.8783545 |
| HPT | 11.80 ± 1.06 | 11.21 ± 0.61 | **0.006** | ** | -0.5841484 |
| **5-0-5 (s)** |  |  |  |  |  |
| CTR | 2.56 ± 0.18 | 2.41 ± 0.15 | **<0.001** | **** | -0.9250099 |
| MPT | 2.52 ± 0.19 | 2.37 ± 0.11 | **<0.001** | **** | -0.9677735 |
| HPT | 2.53 ± 0.16 | 2.37 ± 0.16 | **<0.001** | *** | -0.896262 |
| **YYIR2 (mL.kg^-1^.min^-1^)** |  |  |  |  |  |
| CTR | 50.42 ± 1.86 | 51.57 ± 2.15 | **0.001** | *** | 0.7822737 |
| MPT | 50.36 ± 1.39 | 50.72 ± 1.60 | 0.239 | ns | 0.2434142 |
| HPT | 49.95 ± 1.74 | 50.75 ± 1.93 | **0.020** | * | 0.4839688 |
| **IMTP-50 ms (N)** |  |  |  |  |  |
| CTR | 4151.39 ± 2332.02 | 6165.05 ± 2972.53 | **0.004** | ** | 0.6353037 |
| MPT | 3885.87 ± 1403.55 | 6464.04 ± 3960.13 | **0.004** | ** | 0.6387653 |
| HPT | 4124.24 ± 1558.81 | 7358.33 ± 3047.64 | **<0.001** | **** | 1.127509 |
| **IMTP-100 ms (N)** |  |  |  |  |  |
| CTR | 3957.77 ± 1492.63 | 5802.93 ± 2361.20 | **0.001** | *** | 0.7796678 |
| MPT | 4081.59 ± 1416.17 | 5893.56 ± 3113.04 | **0.007** | ** | 0.6037943 |
| HPT | 4118.61 ± 1481.11 | 6295.06 ± 1810.08 | **<0.001** | **** | 1.1584982 |
| **IMTP-150ms (N)** |  |  |  |  |  |
| CTR | 4358.46 ± 1416.20 | 5263.16 ± 1444.43 | **0.014** | * | 0.5256412 |
| MPT | 3879.40 ± 1118.10 | 5239.44 ± 1526.91 | **<0.001** | *** | 0.9239115 |
| HPT | 4215.05 ± 1170.14 | 5734.56 ± 1067.29 | **<0.001** | **** | 1.037901 |
| **IMTP-200 ms (N)** |  |  |  |  |  |
| CTR | 4358.46 ± 1416.20 | 5263.16 ± 1444.43 | **0.014** | * | 0.5256412 |
| MPT | 3879.40 ± 1118.10 | 5239.44 ± 1526.91 | **<0.001** | *** | 0.9239115 |
| HPT | 4215.05 ± 1170.14 | 5734.56 ± 1067.29 | **<0.001** | **** | 1.037901 |
| **IMTP-250 ms (N)** |  |  |  |  |  |
| CTR | 4115.32 ± 1164.38 | 4806.92 ± 1035.65 | **0.012** | * | 0.5350851 |
| MPT | 3731.97 ± 1060.32 | 4828.03 ± 1080.22 | **<0.001** | *** | 0.8377191 |
| HPT | 3948.42 ± 960.69 | 5096.36 ± 873.89 | **<0.001** | **** | 1.0073882 |
| **IMTP-300 ms (N)** |  |  |  |  |  |
| CTR | 3779.73 ± 854.05 | 4254.74 ± 782.33 | **0.014** | * | 0.5228654 |
| MPT | 3438.32 ± 973.00 | 4449.14 ± 1047.57 | **<0.001** | **** | 0.9778599 |
| HPT | 3582.75 ± 890.88 | 4543.28 ± 798.84 | **<0.001** | *** | 0.7840343 |
| Abbreviations. CTR: control group (n=22); MPT: microdosing plyometric training (n=24); HPT: highdosing plyometric training (n=23).  *: bold values denotes significant differences. | | | | | |
